# Accessory Liver Lobe Attached to the Wall of the Gallbladder

**DOI:** 10.7759/cureus.6113

**Published:** 2019-11-10

**Authors:** Ariel H Park, Tien-Anh Tran, Vladimir Neychev

**Affiliations:** 1 Medicine, University of Central Florida College of Medicine, Orlando, USA; 2 Pathology, AdventHealth Orlando, Orlando, USA; 3 Surgery, University of Central Florida College of Medicine, Orlando, USA

**Keywords:** liver, accessory liver, hepatocellular carcinoma

## Abstract

A 47-year-old woman with a history of known gallstone disease presented with worsening post-prandial right upper abdominal pain radiating to the back, abdominal bloating, and nausea. An ultrasound of the abdomen confirmed the diagnosis of cholelithiasis. During laparoscopic cholecystectomy, an accessory liver lobe attached to the anterior wall of the gallbladder was incidentally found. An accessory liver lobe is a rare anatomical variation that mostly remains clinically asymptomatic. Since hepatocellular carcinoma can rarely develop in an accessory liver lobe, intraoperative complete resection should be considered for both therapeutic and diagnostic purposes.

## Introduction

An accessory liver lobe (AL) is a rare congenital anomaly generally due to either defective or excessive development of the liver [1]. It was first described as a lobe of the liver connected to the main liver via a dense membrane by Morgagni in 1767 [2]. Although it can rarely cause abdominal pain and liver dysfunction, AL mostly remains asymptomatic and is commonly found incidentally during laparotomy, autopsy, and radiologic studies [1]. We report a case of an ectopic AL that was incidentally found to be attached to the anterior surface of the gallbladder wall during laparoscopic cholecystectomy.

## Case presentation

**Figure 1:**
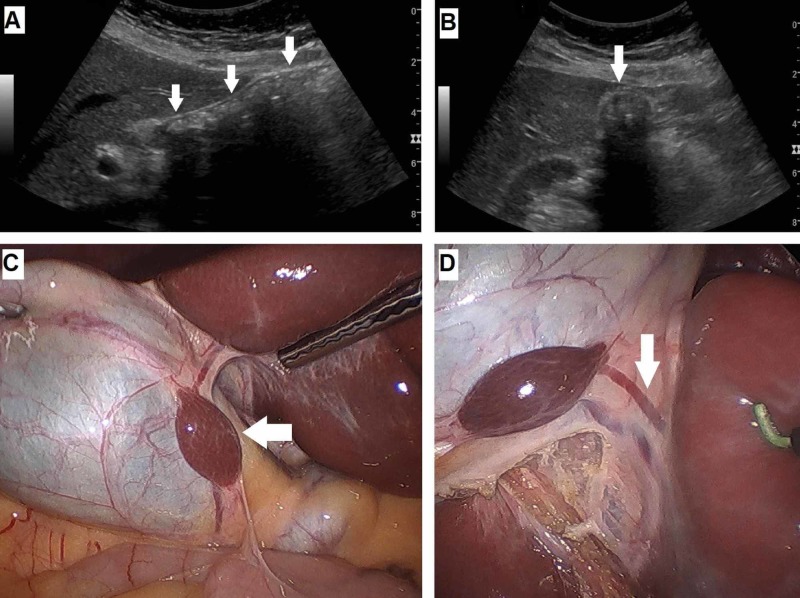
Ultrasound images and intraoperative findings A, Sagittal and B, Transverse ultrasonography images of multiple gallstones represented by reflective echogenic foci within gallbladder lumen with prominent posterior acoustic shadowing (arrows). C, Intraoperative laparoscopic view of the gallbladder retracted over the anterior edge of the liver, revealing an encapsulated accessory liver lobe on the serosal surface of the anteromedial gallbladder wall (arrow). D, Arrow points at a separate vascular pedicle draining the accessory liver lobe well visualized after the mobilization and dissection of the gallbladder from its liver bed.

A 47-year-old woman with a history of known gallstone disease presented to the office with worsening post-prandial right upper abdominal pain radiating to the back, abdominal bloating, and nausea. Her clinical history was significant for hypertension, hyperlipidemia, and tubal ligation. Her vitals were within normal limits, and physical examination showed mild right upper quadrant tenderness without rebound or Murphy’s sign. Laboratory studies including complete blood cell count, liver function tests, and coagulation panel were within normal limits. An ultrasound of the abdomen confirmed the diagnosis of cholelithiasis (Figure 1A, 1B). There was no indication of an AL on the ultrasound images. After a detailed discussion about the biology, natural history, and management options of gallstone disease, including risks and benefits of operative vs non-operative approaches, a decision was made to proceed with an elective laparoscopic cholecystectomy. During the laparoscopic exploration of the Morrison’s space, an approximately 2 cm ellipsoid structure similar in color and consistency to the liver was found attached to the serosal surface of the anteromedial wall of the gallbladder (Figure 1C, 1D).

This finding was consistent with the diagnosis of an AL with its own vascular pedicle. After achieving the critical view of safety, the cystic duct, cystic artery, and the vascular pedicle of the AL were secured with laparoscopic endoclips and transected. The AL was dissected en block with the gallbladder off the liver bed. The patient tolerated and recovered from the procedure well, and she was discharged to home on the day of surgery without complications.

**Figure 2:**
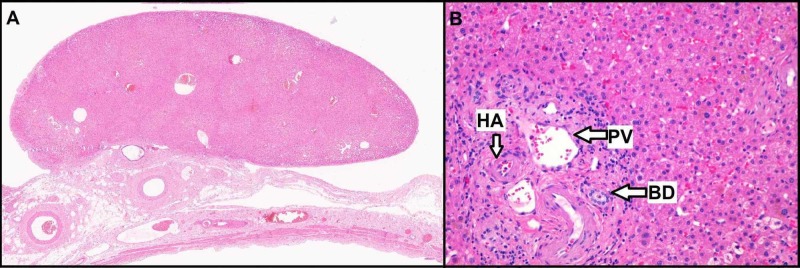
Surgical H&E histopathology A, Low power of the accessory liver lobe attached to the serosal surface of the anterior gallbladder wall. B, High power (x200) of the accessory liver lobe showing portal triad, contains three major structures: portal vein (PV), hepatic artery (HA), and bile ductule (BD).

In addition to several choleliths, gross examination of the gallbladder revealed a 1.4 x 0.9 x 0.4 cm encapsulated fragment of brown soft tissue resembling liver tissue on the serosa (Figure 2A). Histologic examination demonstrated characteristic liver architecture including liver cell cords and sinuses and defined portal tracts containing all three major structures: portal venule, hepatic arteriole, and interlobular bile duct (Figure 2B). The histologic findings supported the diagnosis of an AL. Of note, the AL was located 0.1 cm from the true liver bed of the gallbladder.

## Discussion

An AL is a rare morphologic variation of hepatic tissue usually detected during surgery or autopsy [1]. Most AL result from embryonic heteroplasia but can also rarely occur after trauma or surgery [3]. Two etiological hypotheses have been postulated: (1) a part of the developing liver is entrapped in the septum transversum and subsequently pulled by the weight of intra-abdominal liver, and (2) formation of an AL due to an increased intra-abdominal pressure caused by the development of tunica muscularis recti and enlargement of the liver [4,5].

Different classifications of the AL are documented in the literature. One such classification recognizes three broad types based on the gross anatomical connection of AL to the liver: (1) AL attached to the liver via a stalk; (2) AL representing a tongue-like projection of the anterior edge of the liver without a defined stalk (Riedel’s lobe); and (3) ectopic AL located outside the liver without any connections to the liver [6,7]. Another classification system takes into account the different anatomical variants of the connection between the AL bile duct and the intrahepatic or extrahepatic main biliary tree: (1) AL with a separate accessory lobe duct draining into an intrahepatic bile duct of the liver; (2) AL with a separate accessory lobe duct draining into an extrahepatic bile duct of the liver; and (3) AL with a common capsule with the normal liver and the bile duct draining into an extrahepatic duct [8].

In most cases, AL is located in the infrahepatic location but also can be found at the gastrohepatic ligament, near the umbilicus, adrenal glands, spleen, pancreas, or gallbladder as presented in this case [9]. In addition, ectopic AL has also been reported in extra-abdominal sites such as intrathoracic cavity, vena cava, heart, or lung [10].

The true incidence of all types of AL has been difficult to determine due to its rarity. A laparoscopic observational study revealed the incidence of AL and ectopic liver to be 0.7% [11]. Earlier studies reported the incidence rate of Riedel’s lobe to range broadly from 3.3% to 14.5% [12,13]. Although most AL cases are discovered incidentally during unrelated surgeries, some cases were also found preoperatively on ultrasound or CT images performed for acute abdominal pain or pulmonary symptoms [14,15].

Most patients with an AL remain asymptomatic but rarely can present with acute or recurring abdominal pain, precordial pain, nausea, or vomiting [14]. AL can also lead to complications including torsion, traumatic rupture, infarction, or even more seriously, liver dysfunction or hepatocellular carcinoma formation [9,16]. Extrahepatic AL is believed to have an increased risk of neoplasm due to its incomplete metabolic function and abnormal architecture of arterial supply and venous and biliary drainage [17]. Hepatocellular carcinoma arising in an AL is usually limited to the ectopic AL, and therefore is commonly associated with a favorable prognosis as complete resection of the carcinoma is considered curative [18].

## Conclusions

AL are rare anatomic anomalies that are usually found incidentally during surgery. Although they mostly remain clinically silent, AL has been reportedly associated with the development of hepatocellular carcinoma. Thus, it is important to be aware of, recognize, and resect AL when found preoperatively or incidentally during unrelated surgical procedures.
